# Thyroid cancer burden and economic impact on the Brazilian public health system

**DOI:** 10.20945/2359-3997000000074

**Published:** 2018-10-01

**Authors:** Carolina Castro Porto Silva Janovsky, Marcio Sommer Bittencourt, Maykon Anderson Pires de Novais, Rui M. B. Maciel, Rosa Paula M. Biscolla, Paola Zucchi

**Affiliations:** 1 Universidade Federal de São Paulo Universidade Federal de São Paulo Escola Paulista de Medicina Departamento de Medicina São Paulo SP Brasil Centro de Doenças da Tireoide e Laboratório de Endocrinologia Molecular e Translacional, Divisão de Endocrinologia, Departamento de Medicina, Escola Paulista de Medicina, Universidade Federal de São Paulo (EPM-Unifesp), São Paulo, SP Brasil; 2 Hospital Israelita Albert Einstein Hospital Israelita Albert Einstein Centro de Medicina Preventiva São Paulo SP Brasil Centro de Medicina Preventiva, Hospital Israelita Albert Einstein, São Paulo, SP, Brasil; 3 Universidade de São Paulo Universidade de São Paulo Hospital das Clínicas da Faculdade de Medicina Núcleo de Pesquisa Clínica e Epidemiológica São Paulo SP Brasil Núcleo de Pesquisa Clínica e Epidemiológica, Hospital das Clínicas da Faculdade de Medicina da Universidade de São Paulo (HCFMUSP), São Paulo, SP Brasil; 4 Universidade Federal de São Paulo Universidade Federal de São Paulo Escola Paulista de Medicina Departamento de Medicina São Paulo SP Brasil Divisão de Economia da Saúde, Departamento de Medicina, Escola Paulista de Medicina, Universidade Federal de São Paulo, São Paulo, SP, Brasil

**Keywords:** Thyroid cancer, economic impact, Brazilian public health system, costs, overdiagnosis

## Abstract

**Objective:**

Recent data indicates an increasing incidence of thyroid cancer not accompanied by a proportional increase in mortality, suggesting *overdiagnosis,* which may represent a big public health problem, particularly where resources are scarce. This article aims to describe and evaluate the procedures related to investigation of thyroid nodules and treatment and follow-up of thyroid cancer and the costs for the Brazilian public health system between 2008 and 2015.

**Materials and methods:**

Data on procedures related to investigation of thyroid nodules and treatment/follow-up of thyroid cancer between 2008 and 2015 in Brazil were collected from the Department of Informatics of the Brazilian Unified Health System (Datasus) website.

**Results:**

A statistically significant increase in the use of procedures related to thyroid nodules investigation and thyroid cancer treatment and follow-up was observed in Brazil, though a reduction was noted for procedures related to the treatment of more aggressive thyroid cancer, such as total thyroidectomy with neck dissection and higher radioiodine activities such as 200 and 250 milicuries (mCi). The procedures related to thyroid nodules investigation costs increased by 91% for thyroid ultrasound (p = 0.0003) and 128% in thyroid nodule biopsy (p < 0.001). Costs related to treatment and follow-up related-procedures increased by 120%.

**Conclusion:**

The increase in the incidence of thyroid cancer in Brazil is directly associated with an increased use of diagnostic tools for thyroid nodules, which leads to an upsurge in thyroid cancer treatment and followup-related procedures. These data suggest that substantial resources are being used for diagnosis, treatment and follow-up of a potentially indolent condition.

## INTRODUCTION

Thyroid nodules are a very common condition, found by palpation in 4-7% of the adult population and in more than 50% if an image exam is used (1–4). Despite the general knowledge that thyroid nodules are seldom malignant, about 5%, some studies with necropsies have shown that thyroid cancer may be present in up to 36% of individuals who died from other causes not thyroid-related (5–9).

Indeed, thyroid cancer is the most common endocrine cancer, with an incidence rate of 7.57 per 100,000 women and 1.49 per 100,000 men in Brazil (10), though recent data indicate an increase in incidence worldwide (2,11–14). Interestingly, this increase is not accompanied by a proportional increase in mortality, suggesting the potential diagnosis of early- stage cancer associated with a lower risk of recurrence or the potential occurrence of *overdiagnosis,* which means diagnosing a disease that would never cause symptoms or death during a patient's expected life span (2,15–18).

This phenomenon has been documented in a recent South Korean study, which reported a dramatic rise in the diagnosis of thyroid cancer, reaching epidemic levels, due to the incorporation of a neck ultrasound as part of a routine screening check-up (11). In addition, data from the United States suggest that despite an overall increase in the incidence of thyroid cancers, this phenomenon was more prominent in regions with widely available health care access (15,19,20). Interestingly, such findings seem to be occurring worldwide, leading to increased concern over its public health impact (20).

Recent Brazilian data from the population-based cancer registry (RCBP) has demonstrated a significant increase in the incidence of thyroid cancer in the city of São Paulo from 2008 to 2012 (21). Although this seems to occur throughout the country, the results are more impressive in the southern, southeastern and northeastern regions, where diagnostic tools are more widely available (10,15,22–25).

Since *overdiagnosis* may represent an outsize problem for public health services, it is fundamental to evaluate this phenomenon especially in developing countries, where resources are scarce (22). However, no data on the quantity of procedures performed to investigate a thyroid nodule, treat and follow thyroid cancer patients, as well as its costs for the Brazilian public health system (SUS) are currently available. Therefore, the aim of this study is to describe and discuss the procedures related to investigation of thyroid nodules, treatment and follow-up of thyroid cancer and the direct costs for the Brazilian public health system between 2008 and 2015.

## MATERIALS AND METHODS

A retrospective study was performed using the Department of Informatics of the Unified Health System (Datasus) database (datasus.saude.gov.br) as the main source of information, accessed during the month of December 2016.

**Figure 1:**
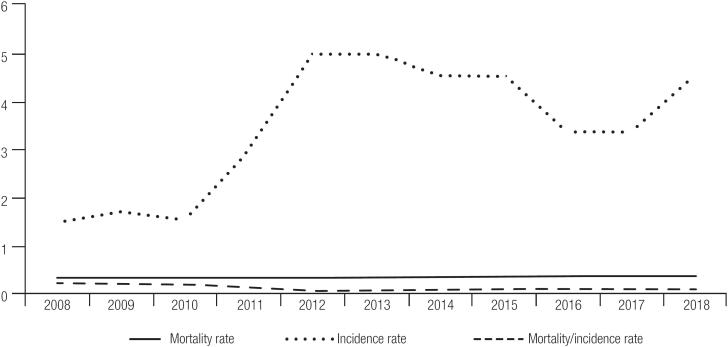
Incidence versus Mortality of Thyroid Cancer (ICD-10: C73) between 2008 and 2018 in Brazil (rate per 100,000 people). Source: Data obtained) from the National Cancer Institute (INCA).

We considered all the procedures present in the algorithm proposed by the national endocrine society for the investigation and management of thyroid nodules (Figure 1 - supplementary material) (26). As thyroid-stimulating hormone TSH measurements are used to investigate thyroid disfunctions and there is not a specific Datasus code for TSH dosage requested for thyroid nodule evaluation, data about TSH measurements were not included.

The quantity and tariffs of the procedures between 2008 and 2015 were accessed through the TABNET link on the Datasus homepage. The data were organized according to the place where the procedure was performed, not by the patient's birthplace. We analyzed data from the entire country, stratified by the five Brazilian regions (south, southeast, northeast, north, and central-west). It was considered only the treatment related-procedures restricted to the 10th edition International Classification of disease code C-73 (‘Thyroid Cancer”) (27).

The tariffs were described in the Brazilian local currency, i.e. *reais.* Considering that the tariff (direct cost) of each procedure for our Public Health System, collected from the SIGTAP (sigtap.datasus.saude.gov.br), hasn't changed since 2008, there was no need for adjusting to the inflation rate.

The thyroid nodule investigation and treatment/ follow-up-related procedures analyzed, as well as each tariff are described in Tables 1 and 2.

**Table 1:** Thyroid Cancer Diagnosis-related Procedures Available at Datasus in December 2016 (sigtap.datasus.saude.gov.br)

Procedure name	Datasus code	Tariff (R$)
Thyroid ultrasound	02.05.02.012-7	24.20
Thyroid FNAB	02.01.01.047-0	23.73
Thyroid scintigraphy	02.08.03.002-6	77.28
Thyroid scintigraphy with suppression and/or stimulus*	02.08.03.003-4	107.30

* Suppression with T3 or T4/stimulus with recombinant human TSH (thyroid-stimulating hormone). FNAB: fine-needle aspiration biopsy.

**Table 2:** Thyroid Cancer Treatment-related Procedures Available at Datasus in December 2016 (sigtap.datasus.saude.gov.br)

Procedure name	Datasus code	Tariff (R$)
Oncologic total thyroidectomy	04.16.03.027-0	2836.30
Total thyroidectomy with neck dissection	04.02.01.005-1	767.77
Total thyroidectomy with neck dissection in oncology	04.16.03.012-2#	1606.86
Trans sternal resection of thyroid cancer	04.16.03.036-0	4186.64
Trans sternal resection of goiter in oncology	04.16.03.005-0*	2618.25
RAI 30 mCi	03.04.09.005-0	443.70
RAI 50 mCi	03.04.09.006-9	614.70
RAI 100 mCi	03.04.09.002-6	1071.90
RAI 150 mCi	03.04.09.001-8	1289.90
RAI 200 mCi	03.04.03.003-4	1471.32
RAI 250 mCi	03.04.09.004-2 03.03.12.001-0	1810.32
WBS	02.08.03.004-2	338.70
Serum thyroglobulin	02.02.06.036-5	15.35

# Code 04.16.03.012-2 was revoked in January 2013. Afterwards, codes 04.16.02.018-6 (unilateral neck dissection in oncology) and 04.16.03.027-0 (total thyroidectomy in oncology) were used to describe this procedure.

* Code 04.16.03.005-0 was revoked in January 2013. Afterwards, code 04.16.03.036-0 (trans sternal resection of thyroid cancer) was used to describe this procedure.

RAI: radioiodine treatment; WBS: whole body scan.

Brazilian population estimates were obtained from the Brazilian Institute of Geography and Statistics (IBGE) website to adjust the quantity of procedures per 100,000 people in each region. The incidence and mortality rate of thyroid cancer was obtained from the latest version of the National Cancer Institute/ Population-based Cancer Registry (INCA/RCBP) database, using the tenth edition of the International Classification of Disease (ICD-10) code “C73” (27).

The statistical analysis was performed with Stata 13.0 (28). To verify the trend of each variable during the period of the study, a Spearman correlation was used. The significance level adopted was 5%.

## RESULTS

In 2008, thyroid cancer incidence rate was 1.51 per 100,000 individuals, rising progressively to 4.57 per 100,000 individuals in 2018 (p = 0.06). The mortality rate rose from 0.30 in 2008 to 0.36 in 2018 (p = 0.004). However, comparing the mortality rate to the incidence rate (mortality rate/incidence rate) there was a negative trend (p = 0.07, rho −0.0674, Figure 1).

**Figure 2:**
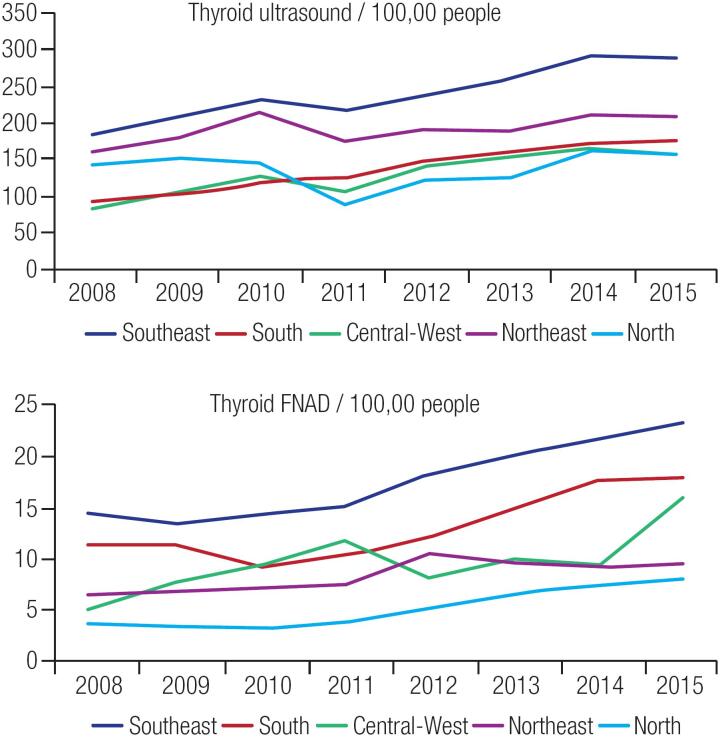
Increase in Numbers of Procedures Related to Thyroid Nodule Investigation between 2008 and 2015 in the Brazilian Public Health System (SUS) by Regions. FNAB: fine-needle aspiration biopsy.

Contributing to this incidence's upsurge, it was observed a statistically significant increase in the number of thyroid nodule investigation tools (thyroid ultrasound and fine-needle aspiration biopsy - FNAB), and treatment/follow-up-related procedures (oncologic total thyroidectomy and radioiodine treatment 100 mCi and 150 mCi) between 2008 and 2015 in Brazil and in all geographic regions (Tables 3, 4 and Figure 2). Data on the increase in treatment- related procedures per geographic region are described in the supplementary material.

**Table 3:** Number of Thyroid Cancer Diagnosis-related Procedures per 100,000 People, between 2008 and 2015 in the Brazilian Public Health System (SUS)

	2008	2009	2010	2011	2012	2013	2014	2015	p
Thyroid ultrasound	154.5	173.2	196.7	173.5	196.1	207.7	232.8	229.6	< 0.001
Thyroid FNAB	10.3	10.1	10.4	11.2	13.5	14.6	15.6	16.9	< 0.001
Thyroid scintigraphy	6.2	5.5	5.6	5.6	5.4	5.5	5.3	4.8	0.001
Thyroid scintigraphy with suppression/stimulus*	0.09	0.06	0.06	0.04	0.04	0.05	0.07	0.08	0.795

* Suppression with T3 or T4/stimulus with recombinant human TSH (thyroid-stimulating hormone).

FNAB: fine-needle aspiration biopsy.

Source: datasus.saude.gov.br.

**Table 4:** Number of Thyroid Cancer Treatment and Follow-up-related Procedures per 100,000 People, between 2008 and 2015 in the Brazilian Public Health System (SUS)

	2008	2009	2010	2011	2012	2013	2014	2015	p
Oncologic TT	0.8	0.9	1.0	1.1	1.2	1.7	1.8	1.9	< 0.001
TT with neck dissection	0.2	0.2	0.2	0.2	0.2	0.2	0.2	0.1	0.027
TT with neck dissection in oncology	0.48	0.56	0.65	0.73	0.84	–	–	–	< 0.001
Trans sternal resection of thyroid cancer*	0.03	0.04	0.03	0.04	0.02	0.02	0.02	0.03	0.046
RAI 30 mCi**	–	–	–	–	–	–	0.06	0.07	< 0.001
RAI 50 mCi**	–	–	–	–	–	–	0.04	0.07	< 0.001
RAI 100 mCi	0.4	0.5	0.7	0.7	0.8	0.9	0.9	0.9	0.027
RAI 150 mCi	0.4	0.5	0.7	0.7	0.8	0.9	0.8	0.8	0.055
RAI 200 mCi	0.3	0.3	0.3	0.3	0.3	0.3	0.3	0.3	0.127
RAI 250 mCi	0.3	0.3	0.3	0.2	0.2	0.2	0.2	0.2	0.007
WBS	3.8	3.7	4.2	4.6	4.4	5.1	4.9	4.9	0.008
Serum thyroglobulin	23.3	27.3	37.3	39.4	38.7	39.8	44.9	48.5	< 0.001

* Data includes codes 04.16.03.005-0 (from 2008 to 2013) and 04.16.03.036-0 (from 2013 to 2016).

** Data available since 2014.

RAI: radioiodine treatment; TT: total thyroidectomy; WBS: whole body scan.

Only the procedures restricted to the IDC: C73 were considered.

Source: Data obtained from datasus.saude.gov.br

The use of thyroid scintigraphy with or without stimulus and/or suppression has reduced during the analyzed period (Table 3).

**Figure 3:**
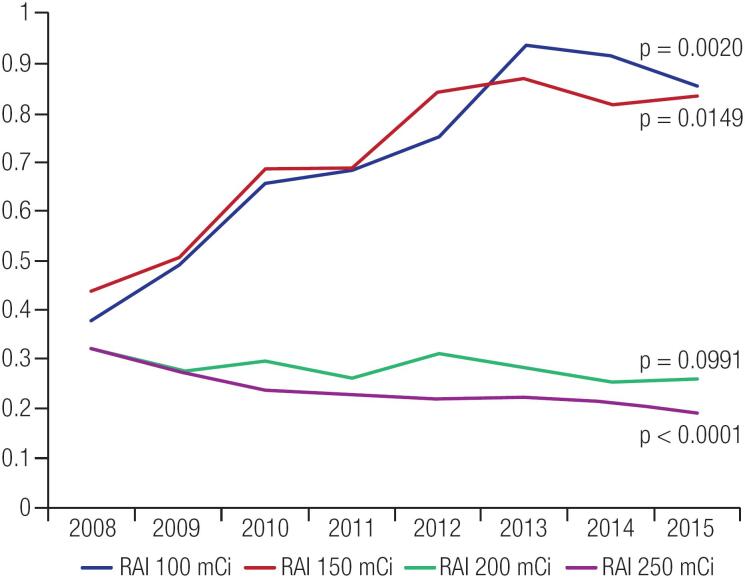
Radioactive Iodine Use per 100,000 People During 2008-2015 in the Brazilian Public Health System (SUS). RAI: radioiodine treatment.

However, the procedures related to more aggressive thyroid cancer treatment reduced significantly during the same period. For example, total thyroidectomy with neck dissection decreased from 0.21 per 100,000 people in 2008 to 0.12 per 100,000 people in 2015 (p = 0.03), while the number of higher radioiodine activities, such as 200 mCi and 250 mCi, both decreased from 0.33 and 0.32 per 100,000 people in 2008, to 0.26 and 0.19 per 100,000 people in 2015, respectively (p = 0.0991 and p < 0.0001). The comparison among levels of radioiodine used for thyroid cancer ablation is shown in Figure 3.

Considering treatment for low-risk patients, an increase in the use of lower doses of RAI (30-50 mCi) for thyroid cancer treatment was noted, although this data is only available since 2014.

**Figure 4:**
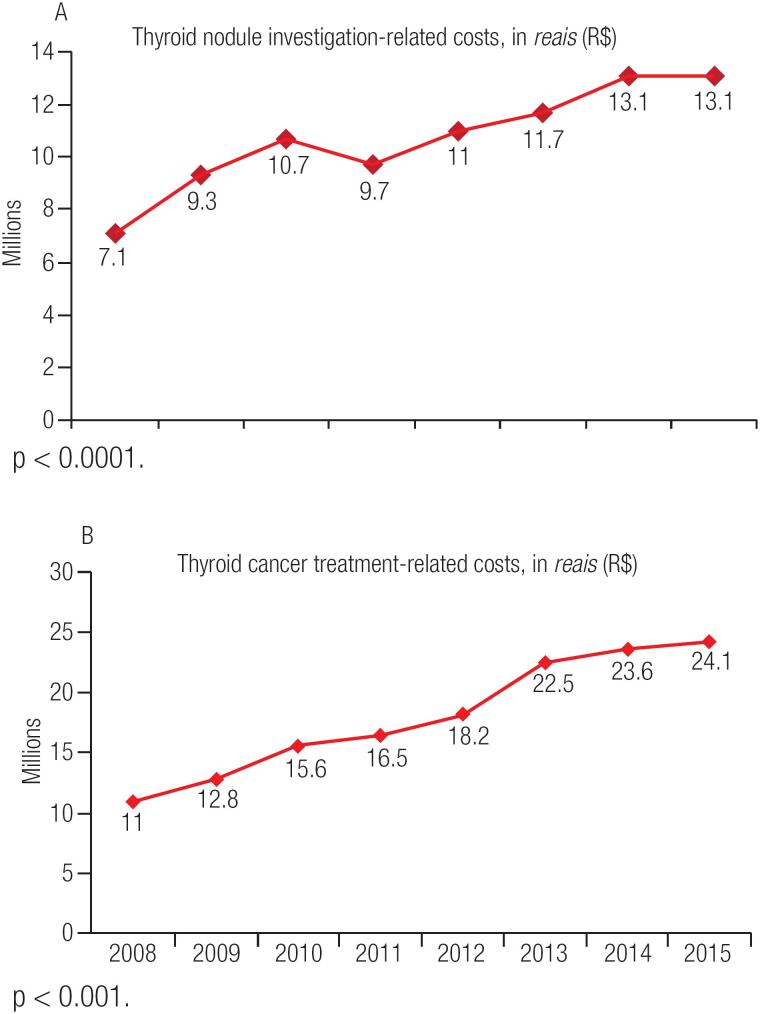
Increase in Costs Related to Thyroid Cancer Diagnosis (**A**) and Treatment (**B**) between 2008 and 2015 in the Brazilian Public Health System.

Regarding direct costs to the Brazilian public health system, a 84% increase in procedures related to thyroid nodule investigation costs was noted in this period. Thyroid ultrasound costs increased by 91% (p = 0.0003), and thyroid nodule biopsy (fine-needle aspiration biopsy) costs increased by 128% (p < 0.001) from 2008 to 2015 (Figure 4A). Similarly, there was a 120% increase in the total costs of treatment-related procedures performed during the same period, mainly due to the increase in the use of oncologic total thyroidectomy, radioiodine activities of 100 and 150 mCi RAI and follow-up procedures (Figure 4B). These procedures (diagnostic and therapeutic) altogether represented an expense of almost 230 million *reais* for the unified health system (SUS) in this 8-year period.

## DISCUSSION

Our study demonstrates an important upsurge in the use of procedures related to thyroid nodule investigation and thyroid cancer treatment and follow-up in Brazil from 2008 to 2015. This overuse of resources has increased the costs of the disease for the Brazilian public health system.

Interestingly, this increase seems to be mostly driven by procedures related to early-stage cancer, as the use of more aggressive surgery and higher-dose radiation therapy has decreased over time. Although this trend might be interpreted as earlier diagnosis due to more intensive use of screening strategies, one would expect a reduction in mortality if this were true. Collectively, this evidence can be interpreted as a potential *overdiagnosis* of cases, which are unlikely to progress to overt or aggressive forms of cancer.

The clinical relevance of a nodule incidentally found by ultrasound is unclear because it probably will not prompt symptoms or neoplastic dissemination (29–31). Indeed, studies have shown that 19% to 68% of the population presents with a thyroid nodule on a neck ultrasound (32,33). In these cases, an early thyroid cancer diagnosis would not improve prognosis, but it may increase the risks related to unnecessary aggressive treatment (31).

To avoid *overdiagnosis* and overuse of resources, health systems in different countries are reconsidering the usefulness of neck ultrasound to screen for thyroid cancer in asymptomatic individuals. In the United Kingdom, only a thyroid specialist is allowed to order neck ultrasounds for patients with thyroid nodules (34). Along the same lines, the American Preventive Service Task Force (USPSTF) recently released its guidelines, in which it strongly recommends against using neck ultrasounds for thyroid cancer screening in asymptomatic people (35). Restricting the use of neck ultrasounds to cases in which palpable nodules are detected by a specialist could be an option for reducing healthcare resource utilization in the Brazilian public health system.

Despite the increase in total thyroidectomies performed during the period of this study, a significant reduction in the use of more complex surgeries, such as total thyroidectomies with neck dissection, was noted. This finding suggests that smaller tumours are being resected, without lymph node metastasis and probably with no important clinical repercussion, which corroborates the hypothesis of *overdiagnosis* (36–38). This concept is reinforced by recent data showing a similar effect with *watchful waiting* compared with surgery when a thyroid nodule is diagnosed as cancer (39,40).

Our results have also documented a significant increase in radioiodine treatment with 100 and 150 mCi, the most common activities prescribed for thyroid cancer patients with intermediate or high risk of recurrence. Lately, national and international guidelines on thyroid cancer management have recommended against the use of radioiodine in low risk of recurrence cases (26,41). Even so, Roman and cols. (42) observed that 30% of the patients with tumours < 1 cm still receive radioiodine activities despite recent guidelines against it (42). In our study, it is not clear if the rise observed in the use of RAI is associated with diagnosis of higher-risk tumours or if clinicians continue to prescribe RAI due to a lack of knowledge or for thyroid remnant ablation. In these cases, the use of RAI could facilitate the use of serum thyroglobulin measurements in the thyroid cancer follow-up (43). Nevertheless, the reduction in prescribing higher RAI activities (200 and 250 mCi) implies that less aggressive cases are being diagnosed.

There was a numerical increase in the use of low radioiodine activities (30-50 mCi), despite the small absolute number of those procedures, as they were only included in the list of authorized procedures by the Brazilian Public Health System in 2014. Recent studies have shown that the benefits of lower RAI doses equal higher doses, such as 100 mCi for low- to intermediate-risk patients, with fewer side effects and reduced costs (44,45). This may be a tendency as low-risk cases are being dignosed. However, the low request for these procedures may indicate that low-risk patients have not received any RAI treatment, which is the most recent standard of care expected for low-risk cases (46,47), or they have received 100 mCi despite the recent literature recommendations (46–49).

The southeastern region of Brazil had the highest increase in number of procedures as well as expenditures related to thyroid cancer diagnosis and treatment. This may have occurred due to the choice of using the Datasus search filter of patients’ treatment place and not their birthplace. It is known that the southeastern region is the richest region and has the largest cancer centres in Brazil, which are referred centres for people from other regions. So the expectation on overuse of resources is higher. Nonetheless, this important upsurge in diagnostic and treatment-related procedures seems to be spread throughout the country, irrespective of region.

From 2010 to 2015, there was a 66% increase in costs related to cancer in Brazil, from 2.1 billion *reais* to 3.5 billion *reais,* according to the National Cancer Institute (INCA). During the 8-year study period, there was also a significant increase in the cost of thyroid nodule investigation and thyroid cancer treatment and follow-up in all Brazilian regions, proportionally higher than what was observed for other types of cancer (106% for thyroid cancer vs 66% for all types of cancer). This excessive expenditure for a potentially indolent disease adds on to the hypothesis of *overdiagnosis.*

Our study, however, must be read within the context of its design. The completeness of the INCA/RCBP database is questionable in some regions. Therefore, the inputs on thyroid cancer incidence may be underestimated. Also, the Datasus database depends on the registry of the procedures performed in each hospital or healthcare service across the country, thus it does not warrant a completely reliable source of information.

Additionally, there are different codes to describe the same procedure, for example, “total thyroidectomy” *versus* “total thyroidectomy in oncology”. This study considered only the code for total thyroidectomy in oncology, which excludes thyroid surgeries for benign diseases. However, the figures may be underestimated, as it is possible that the code for total thyroidectomy (not oncologic) also might have been used to describe surgeries for cancer. Another coding problem is that the code used to describe thyroid FNAB because it is used to describe thyroid as well as parathyroid FNAB. It is known that thyroid tumors are 16 times more common than parathyroid tumors (50). We therefore assumed all FNAB were related to thyroid cancer. Although this may result in a small overestimation of its use, there has been no change in the incidence of parathyroid tumors, and the documented increase over the last eight years is unlikely to have changed if the cases used for parathyroid disease were excluded. Finally, the treatment related-procedure codes that referred to other diseases despite thyroid cancer were disregarded as they might have overestimated the results.

In conclusion, the increasing incidence of thyroid cancer in Brazil seems to be directly associated with the performance of diagnostic procedures, as well as an increase in treatment-related procedures. These data suggest that large resources are allocated for diagnosis and treatment of a potentially indolent condition, which could remain unnoticed throughout one's lifetime. Therefore, it is important that thyroid cancer care be reexamined with a cost-conscious view, providing the best outcome that matters to the patient, relative to the cost of delivering it, especially in developing countries where healthcare resources are scarce.
